# Editorial

**Published:** 1866-04

**Authors:** 


### Editorial.

---

AMERICAN MEDICAL ASSOCIATION.—The next annual meeting of this great national organization will commence on the *first Tuesday* in May, in the city of Baltimore. We are informed that the prospect for a good meeting was never better. Are the delegates appointed to represent the profession of the North-west making themselves ready to attend?

LECTURE FEES IN MEDICAL COLLEGES.—The subject of College lecture fees, seems to have been attracting some attention, more especially from those connected with the Schools in Philadelphia, New York, and Boston. Two or three days since, we saw a printed circular addressed to the Secretary of one of the Colleges in this city, in which it was stated that the Medical Schools in the cities just named had agreed to advance the charge for Lecture fees to one hundred and forty dollars, the previous rates having been, generally, one hundred and five. The circular not only asserted that such advanced rate had been agreed to by the Medical Colleges in those cities, but that it would be so published by them, respectively, in their next annual announcements. The circular was signed by a committee of representatives from the several schools alluded to, and contained a request that the Faculties of the schools to which it was addressed, would communicate their views in relation to the subject, to the chairman of said committee. This forcibly reminded us of a certain class of patients, who go to their physician, and after stating their complaints, gravely announce to him that they have *fully determined* to take certain medicines, and ask him if he does not think they are right. The language of the circular letter shown to us, certainly led to the supposition that so far as the Medical Colleges of Philadelphia, New York, and Boston were concerned, the question of an advance in the rate of lecture fees was settled by a positive agreement to charge, in future, one hundred and forty dollars, which would be an advance of thirty or thirty-five dollars over previous rates. Yet we find in the *Medical and Surgical Reporter*, of Philadelphia, for March 17th, the following editorial paragraph:—

"We have been requested to say that twenty dollars is the highest limit that has been spoken of as the proposed increased rate for tickets in the Medical Colleges. In view of the increased cost of instruction, the amount would not be unreasonable."

How this is to be reconciled with the positive statements of the official circular, we do not know. It would seem to indicate, however, that the question of fees is not yet settled, even in the schools of the principal cities of the Atlantic States. In what used to be called the North-Western States, the lecture fees in the Medical Colleges range from seventy dollars down to nothing. In Cincinnati, for instance, there are three Medical Colleges, one of which charges a lecture fee of $15.00, another $40.00, and another $70.00. In Chicago there are two Colleges, one charging $40.00, with a sixteen weeks' term, and the other, $50.00, with a five months' term.

At Ann Arbor, the medical department of the University of Michigan charges $10.00, as an initiation fee, to be paid but once, and no lecture fees. At Keokuk, the medical department of the University of Iowa charges (if we remember right) an initiation fee of $15.00. These figures are sufficient to show that in this section of the Union, at least, there is neither concert of action among the schools, nor any approach to uniformity of charges for lecture fees.

Whether it is possible to obtain such concert of action as to secure more uniformity in the rate of charges, hereafter, is doubtful. But suppose some concerted action was practicable, either by correspondence, or through the agency of a convention, what constitutes the basis for determining how much the annual lecture fees in a medical college should be? Shall they depend on the actual cost of sustaining a college, with the addition of a fair remuneration to each Professor for the time consumed in giving the instruction required in his department? Shall they be made to depend on the estimated ability of a majority of medical students to pay? Is it desirable to make the cost of medical college instruction so great as to discourage attendance on such institutions, and thereby indirectly encourage the entrance upon the practical duties of the profession without collegiate instruction? Or would it conduce to the best interests of both the profession and the community, to have the taxes upon medical knowledge—or, in other words, the cost of obtaining all the advantages of a thorough course of medical college instruction—as little as possible?

All these are questions requiring a fair and candid consideration in determining the proper rate of lecture fees in medical colleges. The circular to which we have alluded, as well as the paragraph quoted from the *Medical and Surgical Reporter*, rests the proposed increase in lecture fees solely on the ground of increased cost of medical instruction.

That the inflated condition of the currency, and the consequent extraordinary prices for everything that enters into the wants of individuals or families, which has existed during the last three or four years, has not only increased the expenses of those engaged as teachers in medical colleges, but has also increased largely the cost of everything required for their museums and means of illustration, needs no proof. And if the lecture fees in our colleges had been proportionately increased three years since, it would have been in strict consonance with the circumstances of the time. But at this moment we have probably reached the climax of paper currency inflation, with the general tendency of prices already downwards; and regarding the matter in a purely financial aspect, with the history of all previous paper currency inflations as our guide, we should expect that by the time the proposed advance in lecture fees would go into practical operation, the financial condition of the country would be anything but favorable for such a change.

But if the present condition of the currency and prices could be supposed permanent, would it, even then, be beneficial either to the colleges, the profession, or the public, for the medical colleges in our large cities to increase the aggregate cost of attendance on a course of lectures in them? It must be remembered that the lecture fees are but a minor part of the expenses necessarily incurred by the student in attending a college course of instruction. The enormous increase in the prices of board, room rent, fuel, lights, clothing, &c., all come as directly upon him as upon the members of the college faculties; and in most of the large cities the cost of these items has increased threefold within four years. This has already caused a decidedly larger proportion of students to resort to colleges located in smaller towns, where the expenses of board, as well as lecture fees, more nearly corresponded with their pecuniary resources. Let the colleges in Boston, New York, Philadelphia, Cincinnati,

St. Louis, and Chicago, add from twenty to thirty-five dollars more to their annual lecture fees, and they will simply make a corresponding increase in the number of students in the lecture rooms of Concord, Woodstock, Pittsfield, Cleveland, Columbus, Ann Arbor, Keokuk, &c. This will certainly be the result, so long as it remains true that a large majority of those who study medicine are young men of very limited pecuniary resources. If the Faculties of the several schools were the only parties affected by the result predicted, we might regard the matter with indifference. But the interests of the profession at large, and of the whole community, are quite as deeply involved as of those connected with the schools.

If it is true that hospital clinical instruction, directly at the bedside of the sick, constitutes a very important and valuable part of medical education, and renders the student receiving it much better prepared to discharge the practical duties of his profession, then certainly it is desirable that students should be enabled to attend such schools as have ample facilities for that kind of instruction; and every measure that tends to prevent this by inducing them to resort to colleges in small towns where no true clinical instruction can be given, is positively injurious both to the profession and the community.

There are good reasons why medical teaching in large cities with good hospitals should be actually cheaper than in country towns. In the first place, the several Professors reside in the city where the college is located, and in giving their courses of instruction they are neither required to pay traveling expenses, extra board, nor be separated for three or four months each year from their regular field of practice, as is often the case with at least a part of those occupying chairs in country schools. In the second place, the additional reputation, and consequent practice, which is given to a man of talent and industry by a position as teacher in a medical college located in a populous city, more than compensates him for the time actually required to give the course of lectures assigned to him. This is especially true of those occupying the chairs of pathology, practical medicine, surgery, and midwifery. Hence those occupying such positions in a large city could much better afford to give their several courses of instruction free, than to do the same work in a small town, and receive therefor a salary of $1,000 or $1,500 per annum. Physicians and surgeons of the highest order of talent accept with avidity appointments as attendants on public hospitals and dispensaries where their services are to be given entirely gratuitous, simply because the additional reputation and influence gained thereby, more than compensates them for the loss of the time required in the performance of their official duties. We see no reason why the same principle does not apply, with equal force, to those occupying places in public colleges, as to those attending public hospitals. We repeat, therefore, that the Faculties of colleges located in large cities, where all the facilities for acquiring a full and practical medical education are at command, *can afford* to make their *lecture fees* actually less than the Faculties of colleges located in small towns can possibly do. And if this course could be adopted practically, it would speedily concentrate all the medical students of our country in the colleges and hospitals of the larger cities, much to the advantage of the educational interests of the profession. We make these suggestions, not as the representative of any medical college, either in Chicago or elsewhere, but simply as an independent journalist. The lecture fees in the Chicago Medical College, with which we are connected, are higher than in any other medical college west of Ohio and north of St. Louis; and with the exception of the medical department of the University of Michigan, its lecture term is one full month longer. And we presume its Faculty will cheerfully advance still further, both in lecture fees and length of term, whenever neighboring colleges will come up to their present standard.

---

CHICAGO MEDICAL SOCIETY:—DISCUSSION ON THE PATHOLOGY AND TREATMENT OF CHOLERA.—The attention of the Chicago Medical Society, during several of its recent meetings, has been occupied with the subject of Epidemic Cholera. The discussion of the subject was opened by Dr. N. S. DAVIS, the substance of whose remarks are given among the original articles in the present number of the EXAMINER.

The discussion has been participated in, to some extent, by a large proportion of the members of the Society, and has been attended with considerable interest. Dr. JOHN BARTLETT gave a very interesting account of the numerous experiments resorted to in the treatment of cholera patients, in a cholera hospital in Louisville, Ky., during the prevalence of the former epidemic. Among these experiments were hot-air lime baths—injections of various substances into the veins (in one instance after injecting into the veins nearly two quarts of fluid, it began to run from the mouth as fast as injected)—mechanically plugging the anus—application of a very large cup over the epigastrium to prevent vomiting (as mentioned by CELSUS)—the giving of teaspoonful doses of calomel—and in the cases of two children, chloroform was given in doses of a teaspoonful every two hours. One of them died and the other recovered. As might have been expected, under such a series of experimentation, founded on no rational pathological ideas, nearly all the patients died.

Dr. MARGUERAT explained the pathology of cholera, as recently given by Dr. JOHNSON, of London, who claims that the active phenomena of the disease depend directly or indirectly on tonic spasm of the arteries, more especially of the branches of the pulmonary artery, by which the blood is prevented from reaching the pulmonary capillaries freely, and is accumulated in the right cavities of the heart and the large veins connected therewith. The blood, thus shut off from access to the lungs, not only fails to be properly oxygenated and decarbonized, but the veinous fulness extends to the portal vein with its intestinal capillaries, thereby inducing the serous effusions into the alimentary canal. Dr. MARGUERAT thought this theory of arterial spasms not only afforded an explanation of the symptoms of cholera, better than the views presented by Dr. DAVIS, but it also explained why stimulants had so generally proved unavailing or positively injurious in the treatment of the disease, while blood-letting, laxatives, and anæsthetics had often proved beneficial, as shown by the results of such practice, especially in the East Indies. On this theory, not only are all stimulants injurious, but, also, all astringents and opiates that tend simply to suppress the excessive serous evacuations, for such evacuations, in addition to relieving the congested portal system of vessels, also aided in the elimination of the cholera poison. The collapse and death are owing to the failure of blood in the arterial side of the vascular system, and not to the exhaustion of the watery element of the blood by the quantity of discharges. It was also claimed that the theory of spasm was further supported by the alleged fact that, after death, the lungs were generally found pale, bloodless, and compact; the left cavities of the heart empty or nearly so, while the right cavities are over-distended.

Dr. J. P. Ross had listened with much interest to the remarks of Dr. Davis, in opening the discussion, and thought the pathological views presented more plausible than any he had before heard, but inquired whether strychnine did not act more directly on the cerebro-spinal nervous system than the ganglionic, of which the nervo-vascular was a part.

Dr. Lowry, while a student in the Montreal General Hospital, under the direction of Dr. Frazer, saw strychnine used in a considerable number of cases of cholera, during the epidemic of 1854. From the twentieth to the sixteenth of a grain was given in the active stage of cholera every five or ten minutes, until its specific effects began to be visible, and then discontinued. Out of 20 cases thus treated in the hospital, only 4 died.

Dr. Groesbeck stated that during the cholera epidemic of 1854, he gave extract of nux vomica, in the form of pill, with much apparent benefit.

Dr. C. G. Paoli had seen much cholera during the years 1853–4. He regarded the pathological views presented by Drs. Davis and Marguerat as developing nothing new, but were essentially the same as had been promulgated many years since, by different writers. He also claimed that alcoholic stimulants were capable of increasing animal heat, and, therefore, when properly administered, were useful in the treatment of the disease under consideration.

Brief remarks were also made by Drs. Peterson, C. G. Smith, E. L. Holmes, A. Fisher, and D. M. Tucker.

Dr. HOLMES, more especially called the attention of the Society to the efficacy of applying external warmth and irritation, by surrounding the patient with hot bricks wrapped in cloths saturated with mustard water.

Dr. C. G. SMITH had used mustard applications extensively, but not in the mode recommended by Dr. HOLMES. He had also practised, in a few cases, the thorough application of the cold dash or affusion. The patient having been stripped and placed in a tub, had one or two pails full of cold water suddenly dashed over the whole surface, then thoroughly rubbed and wrapped in warm, dry blankets. In patients not anæmic or debilitated previous to the attacks of cholera, he thought the practice beneficial.

Dr. DAVIS, in reply to questions that had been asked during the discussion, stated that the action of strychnine, though manifested plainly on the cerebro-spinal nervous centres, was by no means limited to them, but was exerted efficiently on the tonicity of the whole muscular system of tissues, whether voluntary or involuntary. In relation to the theory of arterial spasm, spoken of by Dr. MARGUERAT, he said it wholly failed to explain the symptoms of the first or initial stage of the disease, as well as many of the important changes during its progress. If contraction of the branches of the pulmonary artery, so as to impede the access of blood to the lungs, constituted an essential pathological condition in cholera, then a sense of suffocation, or want of breath, with disturbance of the heart's action, and fulness of the subclavian and jugular veins, should precede and accompany the initial stage of diarrhœa that ushers in nine out of every ten attacks of cholera. And yet the closest scrutiny has failed to detect the presence of such symptoms in that stage of the disease. It is only when the disease has continued until the stage of collapse is near at hand, that the sense of oppression and want of air, with irregular action of the heart, become prominent symptoms; thus showing their dependency on changes taking place during the progress of the disease, instead of on primary pathological conditions.

Again, he said, contraction or spasm of the pulmonary arteries sufficient to impede the flow of blood to the lungs, ought to disturb the cerebral circulation and functions, through fulness of the descending vena cava and its branches, as directly and speedily as those of the abdominal viscera. Yet there is no other disease of equal rapidity and severity, in which the cerebral functions remain so little disturbed during all the early stage. That mere portal congestion has but little to do with cholera, is logically inferable from the fact that none of the well known causes of such congestion give rise to any of the important phenomena of cholera. Thus, hepatic inflammation, enlargement, and cirrhosis, all cause more or less obstruction to the portal circulation, but induce none of the distinctive symptoms of cholera. In relation to the *post mortem* appearances, he would caution members of the society against the common error of confounding the pathology of a disease with its pathological anatomy; the one related to the nature of the disease, *ab initio*, the other to its final results. He said it was true that in many cases terminating fatally during the stage of collapse, the lungs had been found pale, or indicating the presence, in the pulmonary capillaries, of less blood than natural; but the same appearances were seen quite as often in the mucous membrane of the stomach and intestines, and in the same cases. Yet he presumed no one who had witnessed the amount of discharges from the alimentary canal during life, would have imagined for a moment that the blood had been shut off from that canal by spasm of the vessels supplying it. In regard to inferences to be drawn from the effects of blood-letting and evacuents, he thought them very unreliable. First, because it is by no means established that either blood-letting or other evacuents are generally beneficial in the treatment of cholera. During previous epidemics of cholera, he had bled several patients. In some it appeared to lessen the urgent symptoms, and in others it produced no apparent effect. Some members of the Society, as well as some writers, talk freely of bleeding in the stage of collapse; but according to his observation, bleeding from the arm in true collapse is impracticable, simply because the blood will not flow in sufficient quantity to produce any effect. When it can be made to flow in sufficient quantity, the apparently beneficial effects are more rationally explained by the diminished viscidity of the blood, and the increased absorption by the systemic capillaries, which the direct abstraction of blood from the veins is known to produce, than by any supposable relaxing influence over arterial spasm. In regard to the anæsthetic effects of chloroform, in the stage of collapse, or great exhaustion, he said it must be remembered that the experiments of W. A. HAMMOND, and others, had demonstrated the fact that chloroform, inhaled, directly increased the fulness of the vessels of the brain, which would explain the temporary benefits of that agent in the stage of disease when that organ was being rapidly drained of its ordinary supply of blood.

The Society adjourned, leaving the subject still open for further discussion.

---

MERCY HOSPITAL.—This institution, located on the corner of Calumet Avenue and Twenty-Sixth Street, having been relieved of the pauper class of patients, by the opening of the County Hospital, presents now the best hospital accommodations that can be had in Chicago. It is pleasantly located, its rooms cleanly and well ventilated, and its several departments under the care of able and experienced members of the profession.

The ward set apart for the treatment of diseases of females and lying-in women, is under the care of Prof. W. H. BYFORD; the wards for surgical patients, are under the care of Prof. E. ANDREWS; the wards for medical patients and the apartments for single patients, are under the care of Professors N. S. DAVIS and J. S. JEWELL. Patients laboring under diseases of the eye and ear are as readily admitted and as well treated as any other class. Instead of depending on the care of a corps of hired nurses, the whole internal or domestic affairs of the Hospital are under the immediate care of the Sisters of Mercy, whose faithfulness and zeal in caring for, and sympathizing with, the sick is everywhere acknowledged. Clinical instruction is given in the institution, from 1 to 2 o'clock P.M., on Monday, Tuesday, Thursday and Friday of each week.

DEATHS.—At the last annual meeting of the American Medical Association, we were forcibly impressed with the fact that the men who were in the vigor of manhood, and leading workers in the profession twenty years since, were rapidly disappearing from the stage of action. But that impression has been painfully deepened by the occurrences of the few months which have intervened since the pleasant gathering in Boston.

Of all those who took an active interest in the organization of the American Medical Association, and continued to retain that interest unabated, none were more faithful or more highly esteemed than Drs. Thomas W. Blatchford, of Troy, N. Y.; James Couper, of Newcastle, Del.; L. A. Smith, of Newark, N. J.; and D. L. McGugin, of Keokuk, Iowa. But in quick succession they have closed up their earthly labors and entered upon their heavenly reward.

They were each useful citizens, good Christians, eminent physicians, and strong pillars in the social and professional circles in which they lived. Their familiar faces and cordial greetings will be sadly missed at the coming National gathering in Baltimore, on the first of May. But their memories will long be cherished by all who knew them.

---

DISEASES OF THE EYE AND EAR IN THE COUNTY HOSPITAL.—We learn, with pleasure, that the managers of the Hospital for the County poor in this city, have set apart special wards for patients laboring under diseases of the eye and ear, and have appointed Dr. JOSEPH HILDRETH, formerly Surgeon to the Desmarres Eye and Ear Hospital, to take charge of the same. The appointment is a good one, and will result in having, not only full justice done to that class of poor patients, but will also add a very valuable item to the means for clinical instruction in our city.

---

GRADUATES.—In the list of Graduates at the recent Commencement of Chicago Medical College, in the March number, the name of Wm. E. Turner was accidentally omitted. Dr. Turner graduated with credit to himself and the College.

DISEASE OF THE EYE, CONNECTED WITH A DISEASED TOOTH. —Dr. HILDRETH'S case, published in the original department of this number of the EXAMINER, was previously published in the *Dental Journal*, at Cincinnati, but was furnished to us, with a few additional lines, by the author.

---

LONDON LANCET.—The March number was promptly issued, and contains a continuation of the Lectures on Dyspnœa, by Dr. Hyde Salter; on Puerperal Fever, by Dr. Robert Burnes; and on the Progress of Medicine during the last three centuries, by Dr. W. H. Dickinson; together with the usual variety of interesting and important articles.

---

AMERICAN MEDICAL ASSOCIATION.

The Seventeenth Annual Session will be held in the City of Baltimore, on Tuesday, May 1, 1866.

The following Committees are expected to report:—

On Prize Essays, Dr. Austin Flint, Sr., N.Y., Chairman.

On Quarantine, Dr. Wilson Jewell, Pa., Chairman.

On So-called Spotted Fever, Dr. Jas. J. Levick, Pa., Ch'n.

On Ligature of the Subclavian Artery, Dr. Willard Parker, N.Y., Chairman.

On Tracheotomy in Membranous Croup, Dr. Alex. N. Dougherty, N.J., Chairman.

On Rank of Medical Corps in the Army, Dr. C. S. Tripler, U.S.A., Chairman.

On Rank of Medical Corps in the Navy, Dr. T. L. Smith, N.Y., Chairman.

On Medical Literature, Dr. C. A. Lee, N.Y., Chairman.

On Medical Education, Dr. Samuel D. Gross, Pa., Chairman.

On American Necrology, Dr. C. C. Cox, Md., Chairman.

On Patent Rights and Medical Men, Dr. David Prince, Ill., Chairman.

On Alcohol and its Relations to Man, Dr. Gerard E. Morgan, Md., Chairman.

On Insanity, Dr. Alfred Hitchcock, Mass., Chairman.

On Milk Sickness, Dr. Robert Thompson, Ohio, Chairman.

On the Relation which the Doctrine of the Correlation and Conservation of Forces bears to the Physiological and Pathological Condition of the Human System, Dr. S. L. Loomis, D.C., Chairman.

On the Progress of Medical Science, Dr. Jerome Candee Smith, N.Y., Chairman.

On Diphtheria, Dr. H. D. Holton, Vt., Chairman.

On the Comparative Value of Life in City and Country, Dr. Edw. Jarvis, Mass., Chairman.

On Drainage and Sewerage of Cities in their Influence on Health, Dr. Wilson Jewell, Pa., Chairman.

What Effect has Civilization on the Duration of Human Life, Dr. Augustus A. Gould, Mass., Chairman.

On Disinfectants, Dr. E. M. Hunt, N.J., Chairman

On Compulsory Vaccination, Dr. A. Nelson Bell, N.Y., Ch'n.

On Strangulated Hernia, Dr. W. F. Peck, Iowa, Chairman.

On the Causes and Pathology of Pyæmia, Dr. J. J. Woodward, U.S.A., Chairman.

On the Use of Plaster of Paris in Surgery, Dr. Jas. L. Little, N.Y., Chairman.

On the Etiological and Pathological Relations of Epidemic Erysipelas, Spotted Fever, Diphtheria, and Scarlatina, Dr. N. S. Davis, Ill., Chairman.

On Meteorology, Medical Topography, and Epidemics, Drs. J. C. Weston, Me.; P. A. Stackpole, N.H.; C. L. Allen, Vt.; A. C. Garratt, Mass.; C. W. Parsons, R.I.; B. H. Catlin, Ct.; E. M. Chapman, N.Y.; E. M. Hunt, N.J.; D. Francis Condie, Pa.; T. Antisell, D.C.; O. S. Mahon, Md.; T. M. Logan, Cal.; R. C. Hamill, Ill.; J. W. H. Baker, Iowa; Abm. Sager, Mich.; J. W. Russell, Ohio.

WM. B. ATKINSON.
*Permanent Secretary, Philadelphia.*

---

Mortality for the Month of February.—The following report of the health-officer gives the mortuary statistics for the month of February. From this report it will be seen that the increase in the number of deaths last month over the number in the corresponding month of last year is only 4; the whole number of deaths during last month having been 260, and during the corresponding month of last year 256. It is very gratifying to notice that not a single case of small-pox occurred during last month, while during the month of February of last year there were 17. That dread and ever-increasing disease, to which modern habits of life seem to be leading us more and more, consumption, took off its usual heavy quota, 17, being the largest number of victims to any one disease during the month.

Still, on the whole, the mortality report for last month, when the increased population is taken into consideration, shows a favorable state of health in the city, when compared with previous years, and especially when compared with last month, during which the deaths numbered 293. There is a decrease in the number of those who died over 50 years of age from the number of the corresponding month of last year, of 5; the number over 50 years of age last month, being 23, and of the corresponding month of last year, 28. The number of children under five years of age who died during last month was 117, and the number who died during February of last year, 107.

DISEASES.

The following table shows the causes of death and the number of each:—

| | | | |
|---|---|---|---|
| Accidents, | 8 | Heart Disease, | 6 |
| Brain Fever, | 2 | Hemorrhage, | 2 |
| Bilious Fever, | 1 | Intemperance, | 1 |
| Congestion of Brain, | 1 | Intermittent Fever, | 1 |
| Congestion of Lungs, | 4 | Inflammation of Brain, | 3 |
| Colds, | 6 | Inflammation of Bowels, | 2 |
| Cancer, | 5 | Inflammation of Lungs, | 5 |
| Consumption, | 17 | Lung Fever, | 9 |
| Cramps, | 9 | Nervous Fever, | 1 |
| Croup, | 18 | Rheumatism, | 1 |
| Convulsions, | 11 | Spasms, | 3 |
| Childbirth, | 12 | Suicide, | 1 |
| Cerebro-Spinal Meningitis, | 3 | Stillborn, | 11 |
| Colic, | 1 | Scarlet Fever, | 12 |
| Cholera-Morbus, | 1 | Suffocation, | 3 |
| Congestive Chills, | 3 | Old Age, | 9 |
| Decline, | 2 | Pneumonia, | 4 |
| Diphtheria, | 11 | Paralysis, | 1 |
| Delirium Tremens, | 1 | Phthisis, | 1 |
| Dropsy, | 11 | Typhoid Fever, | 11 |
| Dysentery, | 1 | Teething, | 4 |
| Diarrhœa, | 5 | Whooping-Cough, | 2 |
| Disease of Kidneys, | 1 | Water on Brain, | 2 |
| Disease of Liver, | 3 | Worms, | 1 |
| Diabetes, | 1 | Unknown, | 25 |
| Erysipelas, | 1 | | |
| Fever, | 1 | Total, | 260 |

Ages of the Deceased.—Under 5 years, 107; over 5 and under 10 years' 28; over 10 and under 20, 12; over 20, and under 30, 20; over 30 and under 40, 31; over 40 and under 50, 20; over 50 and under 60, 9; over 60 and under 70, 6; over 70 and under 80, 5; over 80 and under 90, 3; stillborn 11; unknown, 8. Total, 260.

NATIVITIES,

The following table shows the nativity of the deceased:—

| | | | | | |
|---|---|---|---|---|---|
| Chicago, | 142 | Canada, | 4 | Unknown, | 12 |
| Other States, | 33 | Ireland, | 30 | | |
| England, | 8 | Scotland, | 1 | Total | 260 |
| Germany | 27 | Sweden, | 1 | | |
| France, | 1 | Poland, | 1 | | |

COMPARATIVE STATEMENT.

The following is a comparative statement, showing the number of deaths in each division of the city for the months of February in 1865 and 1866:—

| February, 1865. | | February, 1866. | |
|---|---|---|---|
| North Division, | 61 | North Division, | 55 |
| South Division, | 101 | South Division, | 94 |
| West Division, | 94 | West Division, | 111 |
| Total, | 256 | Total, | 260 |

ANNUAL REPORT OF THE INSANE ASYLUM OF CALIFORNIA.—The 13th Annual Report of the State Asylum is before us. It contains no less than four reports from physicians—one from Drs. Whitney and Morse, majority of the Visitors, a minority report from Dr. Hubbard, the report of Dr. Shurtleff, Resident Physician, and that of Dr. Tilden, late Resident Physician. We cannot pretend to analyze these papers, or even to notice them severally. The new building which accommodates 125 female patients, is highly praised as beneficial in itself and also in the relief it gives to the main building, by depleting it of its crowded population. In the majority report of the Visitors, the unfitness of the locality, from the malarious nature of the country and other causes, is appropriately set forth. Dr. Shurtleff is complimented in both reports for his qualifications as Resident Physician. His report is interesting and practical, though it strikes us as decidedly rose-colored in regard to the curative value of the institution. On comparison with thirteen of the Eastern Asylums, the Stocton institution, though for the most part so crowded as to render proper treatment absolutely out of the question, presents a percentage of 51 cures in 14 years, against an average of about 45 per cent. in the Atlantic States. This is a very different result from that which we had been led to expect. If our Asylum can thus take the lead in curativa power without proper accommodations, what would it not accomplish if suitably equipped? Less than five per cent. of the patients discharged as recovered have been returned. This is a very small proportion. Of the deaths in the last year, 28 per cent. were from pulmonary consumption, and the same number precisely from general paralysis. There were 632 inmates at the date of report, 462 of whom were males and 170 females. Admitted during the year 268, viz.: 190 males and 78 females. Increase of insane patients about 50 per year. Ratio of mortality one to eight.—As to the causes in 149 cases, as stated in the commitments, 34 are ascribed to masturbation—more than double the number from any other single cause. Nine cases are allotted to disappointment in love, 13 to pecuniary trouble, 8 to domestic trouble, and 13 to the influence of various other mental afflictions, making 43 in all, or more than one-fourth the whole number, from causes of especial potency on this coast. A sad story is this! No less than 16 cases are set down under the ambiguous head of "religion," and 3 under spiritualism. It is quite refreshing to note even one case of insanity in a female from "absence of husband," though we look in vain for "absence of wife" as a cause. "Disappointment in love" goes harder with men than women, in the proportion of 6 to 3, while "jealousy" crazes two females without perturbing any of the sterner sex. "Meningeal irritation" and "illness" are rather indefinite terms in this relation, and "quinine given to the mother while nursing," is a very unsatisfactory statement. We suppose, however, that under the circumstances in which the commitments are made in various sections of the State, it is not practicable to procure a more technical and rational account of the etiology of insanity.—*Pacific Med. & Surg. Jour.*

---

TREATMENT OF ASIATIC CHOLERA.—Perhaps the most remarkable form of treatment which has been employed in past epidemics of cholera, and reported as being successful, is the strychnia and oleum terebinthinæ treatment, a report of which may be found in the *Transactions of the American Medical Association*, vol. v., page 441, being part of the Report on the Epidemics of Ohio, Indiana, and Michigan, by Dr. G. Mendenhall. The treatment was practised in 1850, in the Commercial Hospital, Cincinnati.

I read from the report referred to:—"The following prescription was first tried (in the Commercial Hospital) by Dr. Howes, one of the resident physicians, under whose administration it was continued by the consent and advice of Prof. Edwards, the attending physician at the time, both of them concurring in its propriety. ℞. Strychniæ gr. ss. ; ol. terebinth., f℥ij. ; mucil. acaciæ, f℥viij. M. Dose—one tablespoonful, to be repeated every half-hour, until the discharges ceased and perfect reaction occurred. In some of the worst cases, the dose was repeated every fifteen minutes. In one case, the amount named in the above prescription was renewed seven times (equal to 3½ grains of strychnia) and given in the course of forty-eight hours. In one case only were the poisonous effects of the strychnia observed; in this case, Ojss. of the mixture (equal to 1½ grains of strychnia) was administered in the course of sixteen hours, and quite severe tetanic spasms were produced, which were, however, relieved in a short time by chloroform inhalations, and the patient recovered

"The number of cases of cholera treated in the hospital during the year was fifty-seven. Seventeen of these were submitted to a treatment consisting mainly of the exhibition of calomel, camphor, morphia, &c. The conditions and results were as follows:—

| | | | |
|---|---|---|---|
| In collapse (pulseless at the wrist), | 8 ; | recovered, | 0 |
| In approaching collapse, | 8 ; | " | 3 |
| In early stage, | 1 ; | " | 1 |
| | 17; | " | 4 |

"Forty cases were submitted to what is called 'strychnia treatment;' the condition and results were as follows:—

| | | | |
|---|---|---|---|
| In collapse (pulseless at the wrist), | 12 ; | recovered, | 2 |
| In approaching collapse, | 20 ; | " | 18 |
| In early stage, | 8 ; | " | 8 |
| | 40 ; | " | 28 |

"Six of the fatal cases reacted perfectly under the strychnia and turpentine, and died of consecutive fever."

In fairness to the "strychnia treatment" I have to state, that all of the accounts that I have met with in regard to it in the hands of other physicians than those of the Commercial Hospital, do not sustain its utility.—*Boston Med. & Surg. Rep.*

---

THE THERAPEUTIC AND PHYSIOLOGICAL ACTION OF DIGITALIS.—1st. That digitalis will stimulate and strengthen a weak heart, and that the weaker the muscular tissues of the heart the safer will be the administration of the medicine.

2d. That in hypertrophied heart it will fail to reduce the pulse either in frequency or strength, and in such cases will prove dangerous.

3d. That in a weak organ, acting because of its weakness with great rapidity, it will reduce the number of its contractions, and, as it were, strengthen or tone them down. To strengthen and quicken the action of a weak, slowly-acting heart, and to reduce the number of the rapid strokes of a feeble heart is according to Dr. Anstie, to do the work of a true stimulant, bringing action up to the normal standard on the one hand, and reducing it to that level on the other.

Thus it appears that the *physiological* action of digitalis is that of a stimulant, in Dr. Anstic's sense of that term; and that in its *therapeutic* properties it is especially useful in cardiac weakness, whether that weakness be accompanied by extremely slow or extremely rapid action. Further its physiological action as a stimulant may be explained by supposing that in the case of the slow heart it improves the molecular arrangement of the sarcous elements, or that it excites the nerve centres from which the nervous power of the heart is derived; and in the case of the weak but rapid heart, it acts by strengthening that regulating or restraining (vital) influence which, while maintaining the activity of the tissues at a normal rate, checks undue and riotous action in the same (Radcliff). Lastly, let me say that as a diuretic it is at once the safest and best we possess, and the dose may vary from ten drops to a half an ounce. This very day I have given nearly half an ounce in fifteen-drop doses every two hours to a child three years old, and by so doing have subdued a rapidly-developing general dropsy, which threatened the little sufferer's life.—*London Med. Times & Gaz.—Boston Med. & Surg. Jour.—Cincinnati Lancet & Observer.*

---

An Italian physician has recently discovered a remedy for certain forms of neuralgia. Attributing the obstinacy of the disease (trifacial neuralgia) to the variations of temperature so frequent in Sicily, he adopted the expedient of covering all the painful parts with a coating of collodion containing a certain portion of hydrochlorate of morphine. This treatment was perfectly successful; the relief was instantaneous and permanent, and the coating fell off in the course of one or two days.—*Medical & Surgical Reporter.*

---

Dr. James P. White, of Buffalo, has sailed for Europe, with the intention of being abroad for eighteen months.

---

M. Jobert de Lamballe, a distinguished surgeon, well known to students of Medicine visiting Paris, is suffering from mental derangement, and is confined in a Lunatic Asylum.

---

Dr. T. G. Thomas has been elected to fill the Chair of Obstetrics in the College of Physicians and Surgeons of New York, rendered vacant by the death of Prof. C. K. Gilman.—*Cincinnati Lancet & Observer.*

